# Upfront surgery versus chemotherapy neoadjuvant in the survival of patients with locally advanced gastric signet-ring-cell adenocarcinoma. A scoping review

**DOI:** 10.3332/ecancer.2025.1843

**Published:** 2025-02-12

**Authors:** Erick R Vásquez-Jaico, Edgar Fermín Yan-Quiroz, Nicol Bonilla-Feria, Mery Nancy Villarreal Gonzalez, Luis Salas Guzmán, Gustavo Adolfo Vásquez-Tirado, Victor Serna-Alarcon

**Affiliations:** 1 School of Medicine, Faculty of Medicine, Antenor Orrego Private University, Trujillo 13008, Perú; 2 Jose Cayetano Heredia EsSalud Regional Hospital, Piura 20002, Perú; 3 Virgen De La Puerta High Complexity Hospital Essalud, Trujillo 13013, Perú; 4 Instituto Regional de Enfermedades Neoplásicas Centro, Junín 12001, Perú; 5 National University of San Marcos, Lima 15001, Perú; a https://orcid.org/0000-0003-4350-7461; b https://orcid.org/0000-0002-9128-4760; c https://orcid.org/0009-0006-3659-7277; d https://orcid.org/0000-0001-7998-8864; e https://orcid.org/0009-0008-2893-272X; f https://orcid.org/0000-0002-2109-6430; g https://orcid.org/0000-0002-9803-6217

**Keywords:** gastric signet ring cell carcinoma, neoadjuvant chemotherapy, upfront surgery, scoping review

## Abstract

**Background:**

Recent research suggests that neoadjuvant chemotherapy is not effective for gastric cancer with signet ring cells.

**Objective:**

The present study performs a scoping review of research that seeks to determine whether neoadjuvant chemotherapy is more effective than upfront surgery in the survival of locally advanced signet ring gastric adenocarcinoma.

**Design:**

Online databases such as Pubmed, scopus and embase were used to identify articles from the last 20 years that used survival, as an initial or secondary outcome variable, after upfront surgery or neoadjuvant chemotherapy as initial treatment in locally advanced gastric signet ring cells adenocarcinoma.

**Results:**

After a systematic selection process, five primary studies were selected that evaluated neoadjuvant chemotherapy compared to primary surgery.

**Conclusion:**

Neoadjuvant chemotherapy does not appear to have greater benefit than initial surgery in gastric adenocarcinoma with locally advanced sign ring cells, it is necessary to define which is the most appropriate qt scheme for adenocarcinoma with sign ring cells, clinical trials type studies are required to improve the evidence. Finally, a national clinical practice guide is required as an interpretative map for the management of gastric cancer which may be appropriate as a first step to know the reality.

## Introduction

Gastric cancer is an important public health problem in Peru, with high incidence and mortality rates. According to the Globocan 2020 database, Peru had an estimated 7,684 new cases of gastric cancer and 5,235 deaths from this disease in 2020. The age-standardised incidence rate of gastric cancer in Peru is 15.4 per 100,000 inhabitants, and the mortality rate is 10.5 per 100,000 inhabitants [1].

Gastric adenocarcinoma with signet ring cells often presents with nonspecific symptoms, which can delay diagnosis. Common clinical manifestations include abdominal pain, nausea, vomiting, early satiety, weight loss and fatigue. Unlike other types of gastric cancer, patients with signet ring cell carcinoma often do not present with a palpable mass or lymphadenopathy, as tumour cells diffusely infiltrate the gastric wall. This can make diagnosis difficult, and the cancer is often diagnosed at an advanced stage [2].

The spread of signet ring cell adenocarcinoma occurs primarily through lymphatic spread to regional lymph nodes, which are frequently involved in gastric cancer. Lymph node metastasis is associated with a higher risk of recurrence and worse survival. Peritoneal seeding is another common route of metastasis in gastric cancer, particularly signet ring cell carcinoma. Tumour cells can spread through the peritoneal cavity and form tumour nodules in the peritoneum or invade nearby organs, causing intestinal obstruction, ascites and other complications [3].

Finally, distant metastasis to the liver, lungs, bones and brain can occur through the bloodstream. The liver is the most common site of distant metastasis in gastric cancer, followed by the lungs and bones. Once metastasised, the prognosis of signet ring cell adenocarcinoma is generally poor, with limited treatment options and reduced survival rates [4].

Neoadjuvant chemotherapy is generally recommended for patients with locally advanced or borderline resectable gastric cancer, defined as tumours that involve adjacent structures or metastasise to regional lymph nodes. Studies have shown that neoadjuvant chemotherapy can improve the R0 resection rate, which refers to complete tumour removal with negative surgical margins [5].

The efficacy of neoadjuvant chemotherapy in adenocarcinoma with signet ring cells remains a topic of debate, as this subtype of gastric cancer has unique clinicopathological characteristics and is associated with a worse prognosis compared to non-signet ring cell carcinoma seal. However, some studies have suggested that neoadjuvant chemotherapy can achieve significant tumour downstaging and improve survival outcomes in patients with signet ring cell carcinoma [6].

There is still debate about the role of neoadjuvant chemotherapy in adenocarcinoma with signet ring cells, and given the importance of this type of cancer in our country, we are considering doing this scoping review, the objective of which is to determine whether neoadjuvant chemotherapy is more effective than upfront surgery in the survival of locally advanced signet ring gastric adenocarcinoma.

## Methods

In this scoping review, the research question was based on the pico model, to evaluate the current status of knowledge in the treatment for patients with locally advanced gastric signet-ring-cell adenocarcinoma (problem), such as upfront surgery (intervention) or neoadjuvant chemotherapy (comparison) influence the survival of this disease (result).

To identify relevant documents, searches were carried out in the following bibliographic databases: PUBMED, EMBASE and SCOPUS. The search strategies were drafted by experienced team members and refined through team discussion. The final strategy is found in Table 1. The results were exported to the Rayyan software for the selection of the final articles. Two reviewers (EYQ and EVJ) examined the publications found; subsequently, the selection was carried out by identifying duplicates and evaluating the titles, abstracts and full text of the potentially relevant articles, conflicts were resolved through the analysis of a third reviewer (VSA).

A Microsoft Excel data collection form was designed to extract the relevant data fields from each included study. Data extraction was performed by the two reviewers independently (EYQ and EVJ).

## Results

A total of 740 articles were collected, of which 660 were from Pubmed, 20 from Scopus and 60 from Embase. 9 duplicates were removed. 731 articles were analyzed, determining 15 articles for analysis with subsequent exclusion of 10 articles that did not meet the PICO criteria. Five articles were included in the present study (Table 2).

## Review

The management of gastric adenocarcinoma with signet ring cells remains controversial in the medical community. Current guidelines offer different approaches, highlighting variability in neoadjuvant treatment and its impact on survival and disease progression [7].

The National Comprehensive Cancer Network guidelines, version 3.2023, suggest that for medically fit patients with locoregional disease and potentially resectable tumours at cT2 or higher and any N, perioperative chemotherapy with the FLOT regimen could be considered. Additionally, surgery as primary treatment would be appropriate for cancer T1b or greater, actively bleeding cancers or when postoperative treatment is preferred, leaving the decision to the surgeon's discretion [8].

On the other hand, the sixth edition of the Japanese gastric cancer guidelines recommends neoadjuvant chemotherapy based on curative resection according to imaging diagnosis, considering it only in cT2-4 patients with bulky nodes. Bulky nodes are defined as at least three perigastric second-level lymph nodes measuring 1.5 cm or larger, or a mass of second-level lymph nodes measuring 3 cm or larger. The advantages of neoadjuvant chemotherapy include an increase in overall survival and disease-free survival, while the disadvantages include the risk of overdiagnosis or underdiagnosis, and the potential for disease progression during chemotherapy [9].

The latest guidelines from the European Society for Medical Oncology indicate that for patients with stage IB-III disease, multimodal treatment is preferred, with neoadjuvant chemotherapy using the FLOT regimen as the standard for those who can tolerate the triple cytotoxic regimen. For those who cannot tolerate it, alternatives such as fluoropyrimidine plus cisplatin or oxaliplatin are recommended [10].

The German, Austrian and Swiss guidelines for the systemic treatment of gastric cancer state that stage IB-III patients should start with perioperative chemotherapy followed by surgery and subsequent chemotherapy. These guidelines, designed with special emphasis on locally advanced and advanced gastric cancer, highlight that the FLOT regimen showed improvements in progression-free survival and overall survival according to the FLOT4 study [11].

The Korean guidelines for gastric cancer from 2022 also suggest potential benefits of neoadjuvant chemotherapy for patients cT2-3 N+ or cT4. Evidence of the benefits of neoadjuvant chemotherapy dates back to the MAGIC trial published in 2006, which compared perioperative chemotherapy with the ECF (Epirubicin + Cisplatin + 5-Fluorouracil) regimen versus surgery alone, finding tumour size reduction and a significant increase in overall survival and progression-free survival [12].

The FLOT4 study from 2019 established the FLOT regimen as a reference for neoadjuvant treatment (*n* = 47), showing improvements in overall survival and disease-free survival compared to the ECF regimen from the MAGIC trial for patients with locally advanced gastric cancer. In this analysis, the FLOT-first group showed favorable overall survival compared with the surgery-first group (HR, 0.416; 95% CI, 0.218–0.794; *p* = 0.008), and 3-year survival rates were 58.7% and 30.9% in the FLOT-first group and surgery-first group followed by chemotherapy (*n* = 269), respectively. However, the analysis of the subgroup of patients with signet ring cell carcinoma did not show significant results (HR 0.74; *p* = 0.7459) [6, 13].

The JCOG501 study published in 2019 evaluated the efficacy of neoadjuvant chemotherapy with S-1 plus cisplatin in patients with type 3 and 4 gastric cancer, where the predominant histological type is diffuse, including signet ring cells, (*n* = 151) compared with gastrectomy plus adjuvant chemotherapy with S-1 (*n* = 149), finding a reduction in operating time (median 240 versus 255 minutes, respectively; *p* = 0.024), but no significant differences in morbidity and mortality (15.8% and 0.7% chemotherapy-first group and surgery-first group and 25.2% and 1.3%, respectively) [14].

The PRODIGY study from 2021 investigated progression-free survival in patients with advanced resectable gastric cancer using the DOS regimen as neoadjuvant treatment (*n* = 266) compared with D2 surgery followed by adjuvant S-1 (*n* = 264), concluding that the DOS regimen is effective and tolerable in Korean patients, observing that neoadjuvant treatment improved progression-free survival versus surgery plus adjuvant therapy (HR: 0.70; 95% CI, 0.52 to 0.95; stratified log-rank *p* = 0.023 although no statistically significant results were found in the subgroup with diffuse gastric cancer [15].

The RESOLVE study published in 2021 evaluated the superiority of the neoadjuvant SOX regimen (*n* = 337) compared to adjuvant CapOx (*n* = 340) and the non-inferiority of adjuvant SOX compared to adjuvant CapOx, concluding that perioperative SOX regimen showed significant clinical improvement in disease-free survival (HR 0.77, 95% CI: 0.61–0.97; Wald *p* = 0.028) [16].

The retrospective analysis by Li *et al* [17] concluded that neoadjuvant chemotherapy (*n* = 36) does not provide a 5-year survival benefit compared with primary surgery plus adjuvant (*n* = 108) in patients with gastric signet ring cell carcinoma, recommending initial surgery as the primary therapy for resectable cases [17]. The 5-year overall survival rates of the neoadjuvant chemotherapy group and surgery-first group were 50.0% and 65.0% (*p* = 0.235), respectively, before propensity score-matched and 50% and 64.7% (*p* = 0.192), respectively, after propensity score-matched. Similar studies by Agnes *et al* [18] and Marino *et al* [19] and Messager *et al* [20] also found that chemotherapy does not significantly impact survival in this subtype of gastric cancer.

Finally, a Brazilian propensity score analysis from 2023 found that patients treated with initial chemotherapy (*n* = 112) showed better overall and disease-free survival compared to those who underwent upfront surgery (*n* = 112), highlighting the need for an individualised approach based on tumour and patient characteristics [21].

In conclusion, although various guidelines and studies support neoadjuvant chemotherapy for gastric adenocarcinoma, the evidence of its specific benefit in the signet ring cell subtype remains limited and contradictory. This underscores the importance of personalised evaluation and clinical judgment in the management of these patients.

## Conflicts of interest

The authors declare that they have no conflicts of interest related to this article.

## Funding

The authors declare that this study received no funding from any public, private or governmental institution.

## Figures and Tables

**Figure 1.**
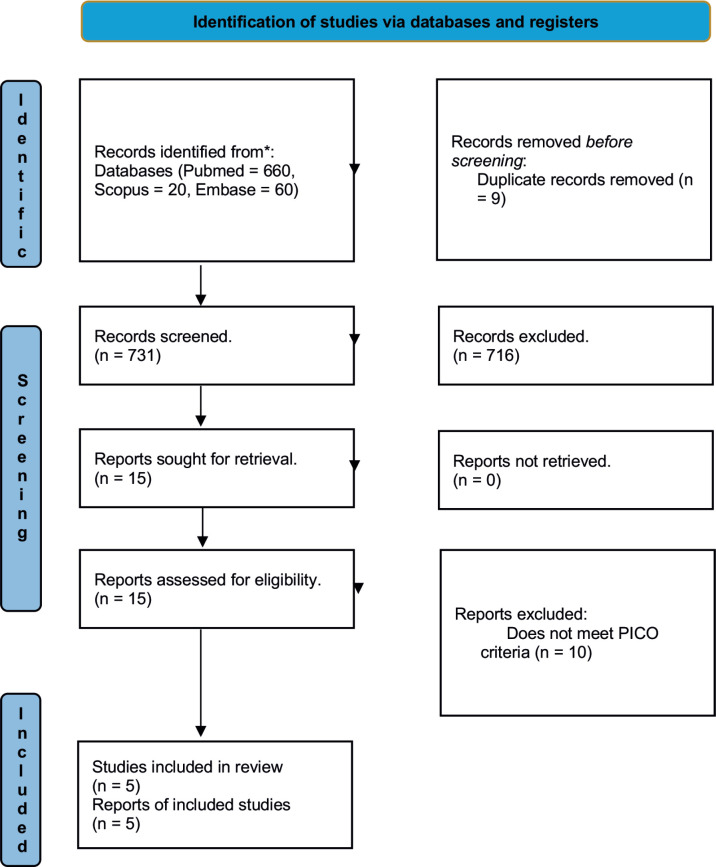
Flowchart showing systematic sequence for included studies.

**Table 1.** Database search strategies.

Data base	Search expression
PUBMED	(Stomach Neoplasms [Mesh] OR "Stomach Neoplasm*"[tw] OR "Gastric Neoplasm*"[tw] OR "Cancer of Stomach"[tw] OR "Stomach Cancer*"[tw] OR "Gastric Cancer*"[tw] OR "Cancer of the Stomach*"[tw] OR "Familial Diffuse Gastric Cancer "[tw] OR "Signet Ring Cell Carcinoma*"[tw] OR "Signet Ring Cell Cancer*"[tw] OR "Signet Ring Cell Neoplasm*"[tw]) AND (Carcinoma, Signet Ring Cell [Mesh] OR “Signet Ring Cell Carcinoma” [tw] OR “Signet ring cell carcinoma of the stomach”[tw] OR " Signet-ring cell carcinoma of the stomach"[tw] OR “Signet-ring cell gastric carcinoma”[tw] OR “Signet ring cell gastric carcinoma”[tw]) AND (“Gastrectomy” [Mesh] OR “Gastrectom*” [tw]) OR/AND (“Lymph Node Excision” [Mesh] OR “Lymph Node Excision*”[tw] OR Lymphadenectom*”[tw] OR “Lymph Node Dissection*”[tw]) AND ("Neoadjuvant therapy"[MeSH Terms] OR Neoadjuvant Therapies[tw] OR Therapy, Neoadjuvant[tw] OR Neoadjuvant Treatment[tw] OR Neoadjuvant Treatments[tw] OR Treatment, Neoadjuvant[tw] OR Neoadjuvant Chemotherapy[tw] OR Chemotherapy, Neoadjuvant[tw] OR Neoadjuvant Chemotherapies[tw] OR Neoadjuvant Chemotherapy Treatment[tw] OR Chemotherapy Treatment, Neoadjuvant[tw] OR Neoadjuvant Chemotherapy Treatments[tw] OR Treatment, Neoadjuvant Chemotherapy[tw] OR Neoadjuvant Systemic Therapy[tw] OR Neoadjuvant Systemic Therapies[tw] OR Systemic Therapy, Neoadjuvant[tw] OR Therapy, Neoadjuvant Systemic[tw] OR Neoadjuvant Systemic Treatment[tw] OR Neoadjuvant Systemic Treatments[tw] OR Systemic Treatment, Neoadjuvant[tw] OR Treatment, Neoadjuvant Systemic)
SCOPUS	TITLE-ABS-KEY("Stomach Neoplasm*" OR "Gastric Neoplasm*" OR "Cancer of Stomach" OR "Stomach Cancer*" OR "Gastric Cancer*" OR "Cancer of the Stomach*" OR "Familial Diffuse Gastric Cancer" OR "Gastric Tumor*" OR "Gastric Tumour*" OR "Stomach Tumor*" OR "Stomach Tumour*" OR "Gastric Malignanc*" OR "Stomach Malignanc*" OR "Gastric Lesion*" OR "Stomach Lesion*" OR "Gastric Mass*" OR "Stomach Mass*") OR TITLE-ABS-KEY(“Signet Ring Cell Carcinoma” OR “Signet ring cell carcinoma of the stomach” OR " Signet-ring cell carcinoma of the stomach" OR “Signet-ring cell gastric carcinoma” OR “Signet ring cell gastric carcinoma”) AND TITLE-ABS-KEY("Gastrectomy" OR “Gastrectom*” OR "Stomach Resection*" OR "Total Gastrectom*" OR "Partial Gastrectom*" OR "Subtotal Gastrectom*" OR "Proximal Gastrectom*" OR "Distal Gastrectom*" OR "Radical Gastrectom*") OR TITLE-ABS-KEY("Lymph Node Excision" OR "Lymph Node Excision*" OR "Lymphadenectom*" OR "Lymph Node Dissection*" OR "Lymph Node Removal*" OR "Lymph Node Biops*") AND TITLE-ABS-KEY("Neoadjuvant therapy" OR "Neoadjuvant Therapies" OR "Neoadjuvant Treatment*" OR "Neoadjuvant Chemotherap*" OR "Neoadjuvant Chemotherapy Treatment*" OR "Neoadjuvant Systemic Therap*" OR "Neoadjuvant Systemic Treatment*")
EMBASE	#1: ′stomach tumor′/exp AND ′signet ring carcinoma′/exp #2: ′lymph node′/exp AND ′gastrectomy′ #3: ′neoadyuvant chemotherapy′

**Table 2.** Characteristics of included studies.

Author (year)	Country	Methods	Sample/specimens	Sample/Signet ring cells			Assessment/Follow up	Conclusion
				Upfront surgery	Neoadyuvant	outcome		
Li *et al* [17]	China	Retrospective Cohort Study	144 patients with with locally advanced signet-ring cell carcinomas of the stomach (cT3/4 and cN any) diagnosed from January 2012 to December 2017	108 (75%)^a^	36 (25%)^a^	The 5-year OS rates in the NAC and surgery-first groups were 50.0% and 65.0% (*p* = 0.235)	Baseline, 5 years after treatment	Neoadjuvant chemotherapy provides no survival benefit in patients with locally advanced gastric signet-ring cell carcinoma.
Agnes *et al* [18]	Italy	Retrospective Cohort Study	269 patients with locally advanced gastric cancer	44 (22.8%)^b^	19 (29.2%)^b^	No differences in disease-free survival (*p* = 0.203)	Baseline, 2 years after treatment	Neoadjuvant therapy had no impact on disease-free survival, disease-specific survival, or the pattern of recurrence in any patients with gastric cancer.
Messager *et al* [20]	France	Retrospective Cohort Study	924 patients with signet-ring cell carcinomas	753 (81.4%)^c^	171 (18.6%)^c^	At a median follow-up of 31.5 months, the median survival was shorter in the perioperative chemotherapy group (12.8 versus 14.0 months, *p* = 0.043).	Baseline, 5 years after treatment	Perioperative chemotherapy provides no survival benefit in patients with gastric SRC.
Marino *et al* [19]	Italy	Retrospective Cohort Study	178 patients with locally advanced gastric cancer	9 (19.1%)^d^	17 (13%)^d^	Patients affected by the intestinal subtype had a better survival when they underwent neoadjuvant chemotherapy compared to patients by the diffuse type	Baseline, 5 years after treatment	NACT could be considered an effective treatment, however, it has to be associated with an excellent surgery, performed by a surgical expert.
Hong* et al* [21]	Brazil	Retrospective Cohort Study	536 patients with gastric cancer	54 (48.2%)^e^	56 (50%)^e^	In univariate and multivariate analyses for disease-free survival and overall survival, after propensity score matching, no difference was found in the Diffuse/mixed versus others histologic type subgroup (HR 1.07 95% CI 0.72–1.59; *p* = 0.739)	Baseline, % yeras after treatment	Preoperative chemotherapy was associated with increased survival in gastric cancer. There was no difference in the postoperative complication rate and mortality compared to upfront surgery.

a Values taken from the study population were signet ring gastric cancer

b The population of the upfront surgery group was 198 patients and the neoadjuvant group 69

c Values taken from the study population were signet ring gastric cancer

d The population of the upfront surgery group was 287 patients and the neoadjuvant group 60

e The study population before propensity score matching was 424 for the upfront surgery group and 112 for chemotherapy. After propensity score matching for both groups was 112 patients

## References

[1] Sung H, Ferlay J, Siegel RL (2021). Global cancer statistics 2020: GLOBOCAN estimates of incidence and mortality worldwide for 36 cancers in 185 countries. CA Cancer J Clin.

[2] Russo AE, Strong VE (2019). Gastric cancer etiology and management in Asia and the West. Annu Rev Med.

[3] López Sala P, Leturia Etxeberria M, Inchausti Iguíñiz E (2023). Adenocarcinoma gástrico: revisión del TNM y de las vías de diseminación. Radiología.

[4] Zhang Y, Lin Y, Duan J (2020). A population-based analysis of distant metastasis in stage IV gastric cancer. Med Sci Monit Int Med J Exp Clin Res.

[5] Ajani JA, D’Amico TA, Almhanna K (2016). Gastric cancer, version 3.2016, NCCN clinical practice guidelines in oncology. J Natl Compr Canc Netw.

[6] Wang K, Ren Y, Ma Z (2019). Docetaxel, oxaliplatin, leucovorin, and 5-fluorouracil (FLOT) as preoperative and postoperative chemotherapy compared with surgery followed by chemotherapy for patients with locally advanced gastric cancer: a propensity score-based analysis. Cancer Manag Res.

[7] Huang ZN, Zheng CY, Wu J (2024). Textbook oncological outcomes and prognosis after curative gastrectomy in advanced gastric cancer: a multicenter study. Eur J Surg Oncol.

[8] NCCN (2024). NCCN Guidelines Version 2.2024 Gastric Cancer.

[9] Japanese Gastric Cancer Association (2023). Japanese gastric cancer treatment guidelines 2021 (6th edition). Gastric Cancer.

[10] Lordick F, Carneiro F, Cascinu S (2022). Gastric cancer: ESMO clinical practice guideline for diagnosis, treatment and follow-up. Ann Oncol.

[11] Lordick F, Al-Batran SE, Arnold D (2024). German, Austrian, and Swiss guidelines for systemic treatment of gastric cancer. Gastric Cancer.

[12] Kim TH, Kim IH, Kang SJ (2023). Korean practice guidelines for gastric cancer 2022: an evidence-based, multidisciplinary approach. J Gastric Cancer.

[13] Cunningham D, Allum WH, Stenning SP (2006). Perioperative chemotherapy versus surgery alone for resectable gastroesophageal cancer. N Engl J Med.

[14] and Terashima M, Stomach Cancer Study Group, Japan Clinical Oncology Group (2019). Randomized phase III trial of gastrectomy with or without neoadjuvant S-1 plus cisplatin for type 4 or large type 3 gastric cancer, the short-term safety and surgical results: Japan Clinical Oncology Group Study (JCOG0501). Gastric Cancer.

[15] Kang YK, Yook JH, Park YK (2021). PRODIGY: a phase III study of neoadjuvant docetaxel, oxaliplatin, and S-1 plus surgery and adjuvant S-1 versus surgery and adjuvant S-1 for resectable advanced gastric cancer. J Clin Oncol.

[16] Zhang X, Liang H, Li Z (2021). Perioperative or postoperative adjuvant oxaliplatin with S-1 versus adjuvant oxaliplatin with capecitabine in patients with locally advanced gastric or gastro-oesophageal junction adenocarcinoma undergoing D2 gastrectomy (RESOLVE): an open-label, superiority and non-inferiority, phase 3 randomised controlled trial. Lancet Oncol.

[17] Li Y, Ma FH, Xue LY (2020). Neoadjuvant chemotherapy vs upfront surgery for gastric signet ring cell carcinoma: a retrospective, propensity score-matched study. World J Gastroenterol.

[18] Agnes A, Biondi A, Laurino A (2020). A detailed analysis of the recurrence timing and pattern after curative surgery in patients undergoing neoadjuvant therapy or upfront surgery for gastric cancer. J Surg Oncol.

[19] Marino E, Graziosi L, Donini A (2021). Neoadjuvant chemotherapy for locally advanced gastric cancer: where we stand; an Italian single center perspective. In Vivo.

[20] Messager M, Lefevre JH, Pichot-Delahaye V (2011). The impact of perioperative chemotherapy on survival in patients with gastric signet ring cell adenocarcinoma: a multicenter comparative study. Ann Surg.

[21] Hong S, Pereira MA, Victor CR (2023). Preoperative chemotherapy versus upfront surgery for advanced gastric cancer: a propensity score matching analysis. ABCD Arq Bras Cir Dig São Paulo.

